# Relationship between IL10 and PD-L1 in Liver Hepatocellular Carcinoma Tissue and Cell Lines

**DOI:** 10.1155/2020/8910183

**Published:** 2020-07-16

**Authors:** Qian Qian, Changping Wu, Jianping Chen, Weibing Wang

**Affiliations:** ^1^Department of Tumor Biological Treatment, Department of Oncology, The Third Affiliated Hospital of Soochow University, 185 Juqian Street, 213003, China; ^2^Chinese People's Liberation Army 904 Hospital, China

## Abstract

**Background:**

Despite the large-scale clinical application of programmed death-ligand 1 (PD-L1) monoclonal antibody, reduction in its clinical response rate has become a gradual problem. As such, use of PD-L1 monoclonal antibody in combination with other anticarcinoma drugs has been the main strategy in improving its efficacy. Interleukin 10 (IL10) is a recognized inflammatory and immunosuppressive factor. Previous studies have suggested that there is a link between PD-L1 and IL10.

**Objective:**

This study was aimed at clarifying the relationship between PD-L1 and IL10 in liver hepatocellular carcinoma (LIHC) and whether IL10 enhances the efficacy of PD-L1 inhibitor.

**Methods:**

Expression levels of PD-L1 and IL10 in carcinoma and adjacent tissues were tested by immunochemistry, Western blotting, and RT-PCR. Survival duration and follow-up data of each patient were recorded. LIHC cell lines Bel7405 and MHCC 97-H were used for *in vitro* experiments. Exogenous IL10 and anti-IL10 were added to cell supernatant. Expression level of PD-L1 in the LIHC cell lines was determined using Western blotting and ELISA. CCK8 and transwell assays were adopted to examine the effect of PD-L1 combined with IL10 on proliferation, invasion, and metastasis of LIHC cells.

**Results:**

The survival period of patients with low expression of IL10 was longer than that of patients with high expression (*P* = 0.01). Overexpression of PD-L1 increased the IL10 and Met levels in LIHC tissues and cell lines. IL10 downregulated the expression level of PD-L1 and enhanced the efficacy of crizotinib via the Met signaling pathway in the LIHC cells.

**Conclusions:**

A combination of IL10 and PD-L1 inhibitor holds great promise as an effective treatment for LIHC.

## 1. Introduction

Primary liver carcinoma ranks second among the top leading cancers with high mortality globally. Primary liver carcinoma is divided into liver hepatocellular carcinoma (LIHC), intrahepatic cholangiocarcinoma, hepatocellular bile duct carcinoma, and fibrous liver carcinoma based on histological type. LIHC accounts for approximately 90% of primary liver carcinoma. Prevalence of liver carcinoma varies across regions. Central Asia, South Asia, Northern Europe, and East China have the lowest incidence of LIHC while Western Europe, South Africa, East Asia, Southeast Asia, and West Africa have the highest incidence rates. In 2012, more than 782,000 new cases of primary liver carcinoma were reported worldwide. This accounted for 5.6% of all carcinomas worldwide ranking sixth among all carcinomas. Among the top five carcinomas were lung carcinoma, female breast carcinoma, gastrointestinal carcinoma, prostatic carcinoma, and esophageal carcinoma. In the same year, 746,000 people died of liver carcinoma worldwide. This accounted for 9.1% of all deaths in the same period. In China, incidence and mortality rates of liver carcinoma are much higher than the global average. This has been attributed to the widespread infection of chronic hepatitis B virus and long-term intake of aflatoxin-containing foods [1–5]. Surgical resection is the most effective way to treat liver carcinoma. Radiofrequency ablation, chemotherapy, and biotherapy are also alternative treatments for liver carcinoma. However, patients diagnosed with advanced liver carcinoma are not fit for surgery. They are mainly treated with radiotherapy and chemotherapy. Cognizant to this, new treatment strategies and drug therapies are urgently needed to improve the quality of life of patients diagnosed with advanced liver carcinoma.

Programmed death-ligand 1 (PD-L1) is a transmembrane protein that is widely expressed in many types of tumor cells. PD-L1 in combination with PD-1 (programmed death-1) receptor on T cells can inhibit T cell activation [6, 7] thereby promoting immune escape and tumorigenesis [8]. Currently, several PD-L1 inhibitors are available to treat some tumors with good treatment effects [9–11]. However, drug resistance limits the therapeutic benefits of a single PD-L1 inhibitor [12–14]. As such, a combination of PD-L1 inhibitors and other treatments has been proposed to reduce drug resistance rate hence improving the clinical response rate [15–20]. For instance, PD-L1 inhibitors combined with immune factors have a stronger anticarcinoma effect than PD-L1 inhibitors used alone [21–23].

Interleukin 10 (IL10) is a multifunctional cytokine produced in multiple cells. It has been found to regulate cell growth and differentiation. It is also an inflammatory and immunosuppressive factor. Previously, IL10 was found to be a negative immunomodulatory factor that inhibits inflammation and immune response promoting the occurrence and progression of tumors [24, 25]. Evidence from several studies has suggested that IL10 can activate immune cells, activate immune functions, and inhibit the occurrence and progression of tumors under specific microenvironments [26–28]. Furthermore, a connection between PD-L1 and IL10 under specific cellular contexts has been proposed [29–33]. However, this connection has not been experimentally established. Here, we investigated the relationship between PD-L1 and IL10. We also investigated the clinical benefits of a combination of IL10 and PD-L1 inhibitors in LIHC.

**Figure 1:**
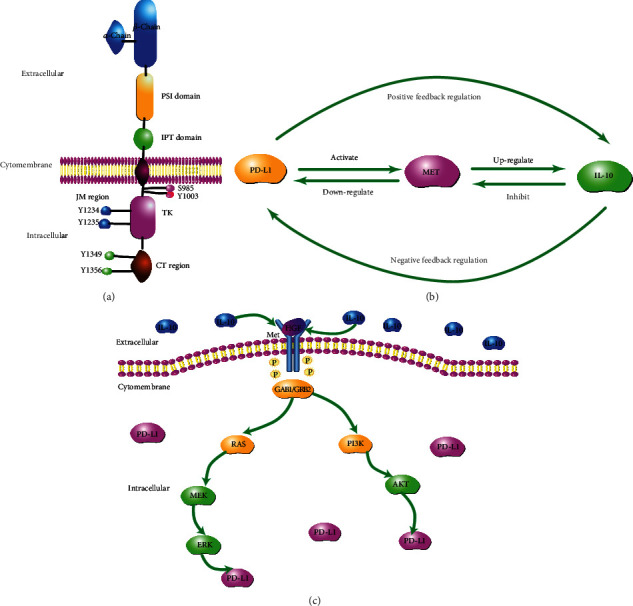
Hand-drawn picture of interactions between PD-L1, Met, and IL10. (a) Met protein structure: Met is a heterodimer consisting of an extracellular alpha chain, and a beta chain that spans the membrane. The Met receptor has three functionally distinct domains, including the extracellular, transmembrane, and intracellular domain. The intracellular domain functions in three channels, including a region adjacent to the membrane-proximal intracellular domain, a tyrosine kinase catalytic structure with tyrosine kinase activity domain, and a C-terminal domain that interacts with a variety of downstream signaling molecules. (b) Dynamic interactions between PD-L1, Met, and IL10. The overexpression of PD-L1 upregulates the expression of IL10 in LIHC via a positive feedback loop while IL10 downregulates the expression of PD-L1 in LIHC via a negative feedback loop. (c) IL10 acts on the HGF (hepatocyte growth factor)/Met signaling pathway, thereby affecting its downstream Akt and MAPK signaling pathway before downregulating the PD-L1 expression.

Met is a receptor for hepatocyte growth factor (HGF) and a tyrosine kinase, which is generally expressed in epithelial cell tissues. HGF/Met signaling pathway has been implicated in diverse physiological and pathological processes [34, 35] (Figure 1(a)).

In this study, we postulated that upregulation of PD-L1 will increase IL10 expression in LIHC cell lines and tissues via a positive feedback loop. We found that IL10 decreased the expression level of PD-L1 in LIHC cells via a negative feedback loop. Met was found to be a key player in the connection between PD-L1 and IL10 (Figures 1(b) and 1(c)). Thus, a combination of IL10 and PD-L1 inhibitor holds great promise as an effective treatment for LIHC.

## 2. Material and Methods

### 2.1. Bioinformatics Analysis

Bioinformatics analysis was performed using TCGA database (http://www.cancer.gov/). The correlation between IL10 and PD-L1 in LIHC as well as the association between IL10 and survival duration was analyzed.

### 2.2. Patients' Information and Tissue Collection

Carcinoma tissues, adjacent tissues (less than 2 cm away from carcinoma tissues), and normal tissues were collected from 100 patients who underwent tumor surgery at the Third Affiliated Hospital of Soochow University between 2013 and 2018. Part of the freshly excised tissue was kept in a liquid nitrogen for further experiments, and the remaining tissue was sectioned into paraffin blocks. None of the patients had received radiotherapy, chemotherapy, or targeted treatment before surgery. The patients comprised 55 males and 45 females aged between 45 and 82 years. Among them, 68 patients were aged above 60 years while the remaining 32 were below 60 years old. 50 patients had highly differentiated tumors while the remaining 50 had medium or lowly differentiated tumors. Based on TNM staging (tumor, node, metastasis), 28 patients were in T1/T2 stage while the remaining 72 patients were in T3/T4 stage. In the same line, 52 patients were in N0 stage while the remaining 48 were in N1 stage. A total of 38 patients had carcinoma in the left lobe of the liver while the remaining 62 had it in the right lobe of the liver.

### 2.3. Patient Follow-Up

The liver function, abdominal B-ultrasound, alpha fetoprotein, and prothrombin levels were reviewed every three to six months after surgery. The survival time of each patient was also recorded. During the follow-up period, all patients were treated with regular liver protective medicine only. They were not given other types of anticancer treatments, including chemotherapy, radiotherapy, or targeted therapy.

### 2.4. Immunochemistry

Immunochemistry was performed to compare differences in PD-L1, IL10, and CD8 levels between liver carcinoma and adjacent tissues. Surgically obtained tumor tissue was embedded in paraffin and then cut into 5 mm sections. The sections were dewaxed and rehydrated for immunohistochemical staining with the corresponding antibodies. The detailed process and scoring criteria are presented in supplementary materials (Table S1).

### 2.5. Quantitative Real-Time PCR

The expression of PD-L1, IL10, and Met mRNA in carcinoma, adjacent, and normal tissues was determined using quantitative PCR. RNA was extracted from the liver tissues using Trizol reagent. The quality and quantity of RNA were determined using a NanoDrop™ 3300 fluorospectrometer. It was then reverse transcribed into cDNA using a RT-PCR kit from Takara company (Tokyo, Japan) and used for PCR amplification. Primers were designed and provided by Shanghai Sangon Company (Table S2). The specific process of RT-PCR is presented in supplementary materials.

### 2.6. Cell Culture

LIHC cell lines Bel7405 and MHCC 97-H were used for *in vitro* studies. Exogenous IL10 and anti-IL10 were added to LIHC cell lines to assess the changes in PD-L1 expression. Details of the culture process are presented in supplementary materials.

### 2.7. Construction of siRNA

Both siMet and siPD-L1 of Bel7405 and MHCC 97-H cell lines were designed by GenePharma Company (Shanghai, China). The details of this process are presented in supplementary materials. siRNA sequences are presented in Table S3.

### 2.8. Overexpression of PD-L1

LIHC cell lines overexpressing PD-L1 were established by lentiviral transfection at the Cell Research Center of the Third Affiliated Hospital of Soochow University. They are abbreviated as LV-PD-L1 in this paper.

### 2.9. Western Blotting and ELISA

Western blotting and enzyme-linked immunosorbent assay (ELISA) were used to evaluate the changes in PD-L1 expression level in LIHC cell lines after introduction of exogenous IL10 and anti-IL10 in the culture. The expression of downstream targets of the Met signaling pathway was also tested by Western blotting (Met, phospho-Met, Akt, phospho-Akt, Mek, phospho-Mek, erk, and phospho-erk). Differences in PD-L1 protein expression when crizotinib was used alone or as a combination with IL10 were compared using Western blotting. The expression levels of IL10 in LV-PD-L1 and siPD-L1 cell lines were determined by Western blotting and ELISA. Similarly, expression levels of PD-L1, IL10, and Met in liver carcinoma tissues, adjacent tissues, and normal tissues were determined using Western blotting. Details of Western blotting and ELISA experiments are presented in supplementary materials.

### 2.10. CCK8 and Transwell Assays

Cell Counting Kit8 (CCK8) and transwell assays were used to determine the effects of crizotinib alone or in combination with IL10 on the proliferation, invasion, and metastasis ability of Bel7405 and MHCC 97-H cell lines (the concentration of IL10 was 1 mg/ml and crizotinib was 1 *μ*M). After different concentrations of IL10 and crizotinib were added to the cell supernatant, CCK8 and transwell were adopted to test the relationship between drug concentration and proliferation, invasion, and migration ability. Similar experiments were performed when Met gene was knocked down. Details of the experimental processes are provided in supplementary materials.

### 2.11. Statistical Analysis

The chi-squared (*χ*^2^) test was used to compare the differences between groups and analyze the correlation between PD-L1 and IL10 and with clinical pathology. Western blotting bands were analyzed using ImageJ software. The Kaplan-Meier curve was used to analyze the survival time of the 100 LIHC patients. The rest of the data were analyzed using SPSS software version 25.0 and GraphPad software version 5.0. All pictures were processed with Photoshop software version 5.0. *P* values less than 0.05 (*P* < 0.05) were considered statistically significant.

## 3. Results

### 3.1. Patient Characteristics and Immunochemistry

**Figure 2:**
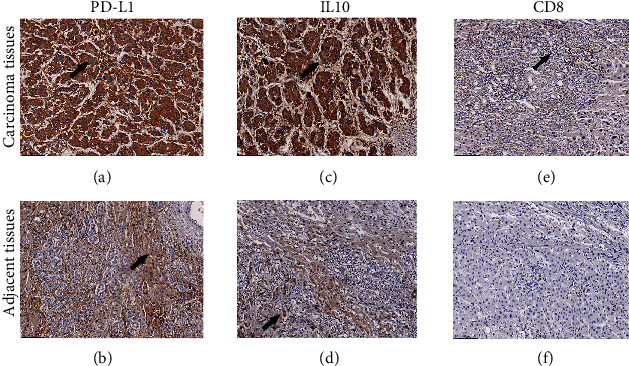
Expression of PD-L1, IL10, and CD8 in LIHC tissues: (a, b) Representative images of immunohistochemical staining with anti-PD-L1 in LIHC tissues (a) and adjacent tissues (b); (c, d) representative images of anti-IL10 in LIHC tissues (c) and adjacent tissues (d); (e, f) representative images of anti-CD8 in LIHC tissues (e) and adjacent tissues (f). The arrows are for positive regions. Magnification: ×200.

The details of patients enrolled in the study are listed in Tables 1 and 2. PD-L1 was detected on the cytomembrane and cytoplasm (Figures 2(a) and 2(b)) while IL10 was mainly expressed in the cytoplasm (Figures 2(c) and 2(d)). CD8 was mainly located on the surface of the T lymphocyte membrane (Figures 2(e) and 2(f)). Patients with lower age (*P* = 0.048), high tumor differentiation (*P* = 0.001), T1/T2 staging (*P* < 0.001), low IL10 expression (*P* < 0.001), and low CD8 expression (*P* = 0.023) exhibited lower PD-L1 expression levels. Gender (*P* = 0.562), N grading (*P* = 1.000), tumor size (*P* = 0.696), and tumor location (*P* = 0.096) were not associated with changes in PD-L1 expression. Patients with lower age (*P* <0.001), T1/T2 grading (*P* < 0.001), N0 grading (*P* < 0.001), tumor size less than 5 cm (*P* < 0.001), and low PD-L1 expression (*P* < 0.001) presented with lower IL10 expression levels. There was no association between gender (*P* = 0.178), tumor differentiation (*P* = 0.137), tumor location (*P* = 0.500), and IL10 expression.

### 3.2. Expression Levels of PD-L1 and IL10 in Adjacent Tissues Were Lower Than in Carcinoma Tissues

**Figure 3:**
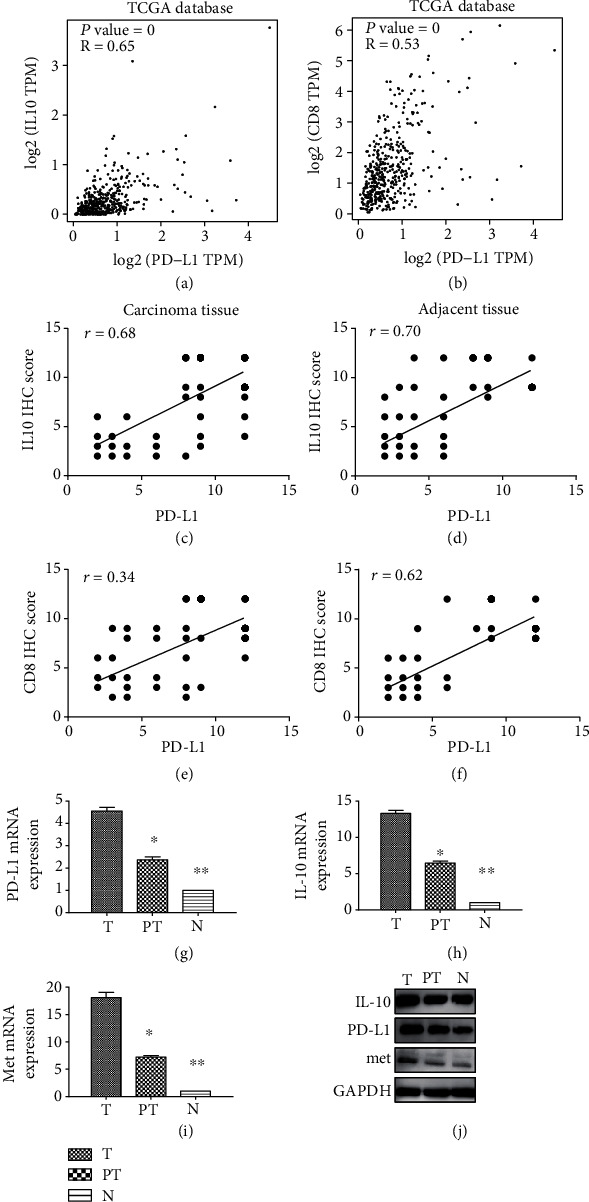
Relationship between the expression levels of PD-L1 IL10 and CD8 and comparison of PD-L1, IL10, and Met in LIHC: (a) Correlation analysis of PD-L1 and IL10 gene expression in LIHC according to TCGA database; (b) correlation analysis of PD-L1 and CD8 gene expression in LIHC according to TCGA database; (c, d) Pearson correlation analysis of expression level of PD-L1 and IL10 in carcinoma tissues (c) and adjacent tissues (d); (e, f) Pearson correlation analysis of expression level of PD-L1 and CD8 in carcinoma tissues (c) and adjacent tissues (d); (g–j) comparison of IL10, PD-L1, and Met mRNA expression levels in carcinoma tissues, adjacent tissues, and normal tissues by quantitative real-time PCR and Western blots. *P* ≤ 0.05 was considered statistically significant. T: carcinoma tissues; PT: adjacent tissues; N: normal tissues. ^∗^*P* < 0.05 compared with carcinoma tissues; ^∗∗^*P* < 0.05 compared with adjacent tissues.

According to immunochemistry analysis, PD-L1 was highly expressed in carcinoma tissues from 72 patients and adjacent tissues from 28 patients. Comparatively, IL10 was highly expressed in carcinoma tissues from 67 patients and adjacent tissues from 35 patients. mRNA and protein levels of PD-L1 (*P* < 0.001) and IL10 (*P* < 0.001) were higher in carcinoma tissues than in adjacent tissues (Tables 3 and 4). Analysis performed on TCGA database revealed that PD-L1 and IL10 were positively correlated at the genetic level (Figure 3(a)). Furthermore, a positive correlation was found between PD-L1 and CD8 expression (Figure 3(b)). Results of the Pearson correlation analysis from our study were consistent with those obtained from bioinformatics analyses (Figures 3(c)–3(f)). Moreover, PD-L1, IL10, and Met were lower in adjacent and normal tissues than in carcinoma tissues both at mRNA and protein levels (Figures 3(g)–3(j)).

### 3.3. PD-L1/IL10 Expression Level Affected Patients' Survival Period

**Figure 4:**
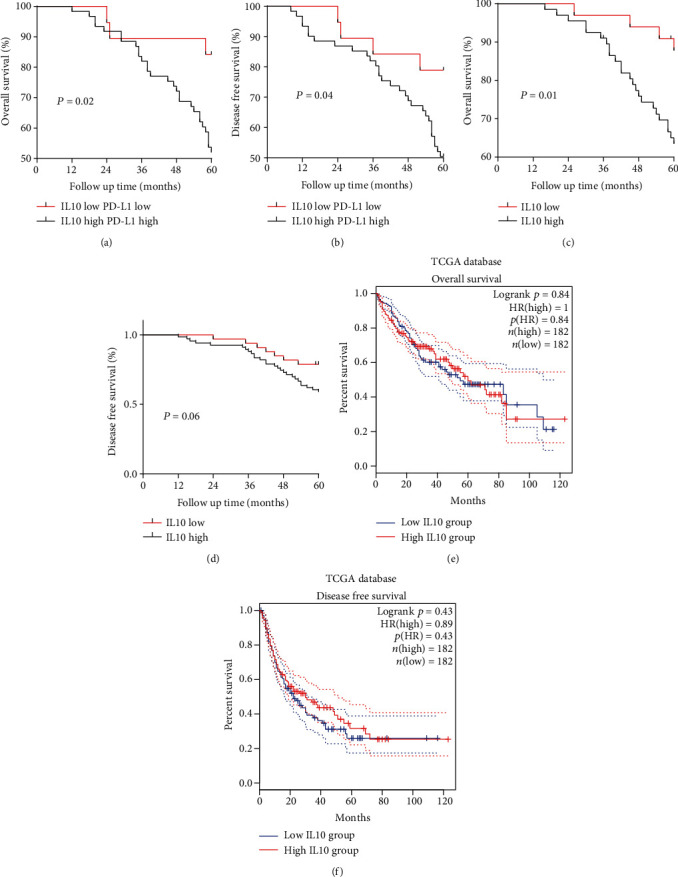
Prognosis according to the PD-L1 and IL10 expression levels in patients with LIHC: (a, b) OS and DFS rates of patients in relation to the PD-L1 and IL10 expression status; (c, d) overall survival (OS) and disease-free survival (DFS) of patients with LIHC in relation to IL10 expression status; (e, f) overall survival (OS) and disease-free survival (DFS) according to IL10 expression of patients with LIHC according to TCGA database.

Analysis of the five-year survival duration of surgical patients revealed that patients with high expression of PD-L1 and IL10 had shorter overall survival (OS) (*P* = 0.04) and disease-free survival (DFS) (*P* = 0.02) than those with low expression of PD-L1 and IL10 (Figures 4(a) and 4(b)). The OS of patients with low expression of IL10 was longer than that of patients with high expression (*P* = 0.01). However, there were no significant differences in the DFS of these patients (*P* = 0.06) (Figures 4(c) and 4(d)). These results contradicted those obtained from TCGA database which showed that the expression level of IL10 did not affect the OS and DFS of the patients (Figures 4(e) and 4(f)).

### 3.4. Correlation between IL10 and PD-L1 Expression in LIHC Cell Lines

**Figure 5:**
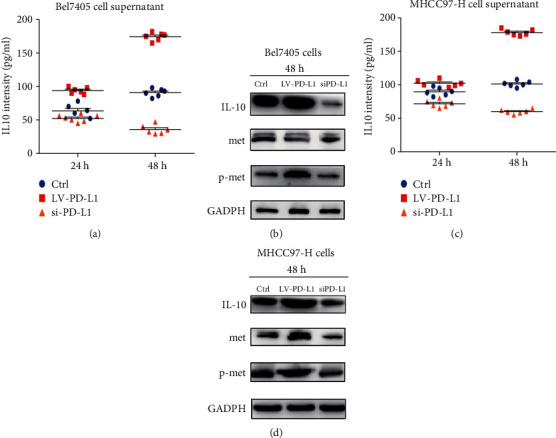
Effects of PD-L1 knockdown and overexpression on the expression levels of IL10, Met, and phosphor-Met in LIHC cell lines. Bel7405 and MHCC 97-H cells (c, d) were transfected with PD-L1 siRNA to knock down the expression of PD-L1 (siPD-L1), and with lentivirus to overexpress PD-L1 (LV-PD-L1). Cells without any transfection were used as the control. The IL10 levels were detected by ELISA (a, c). The levels of IL10, Met, and p-Met were measured by Western blots. GADPH was used as the loading control (b, d).

Upregulation of PD-L1 increased the expression of IL10 and vice versa, in the two LIHC cell lines. The Met signaling pathway was involved in this process (Figures 5(a)–5(d)).

**Figure 6:**
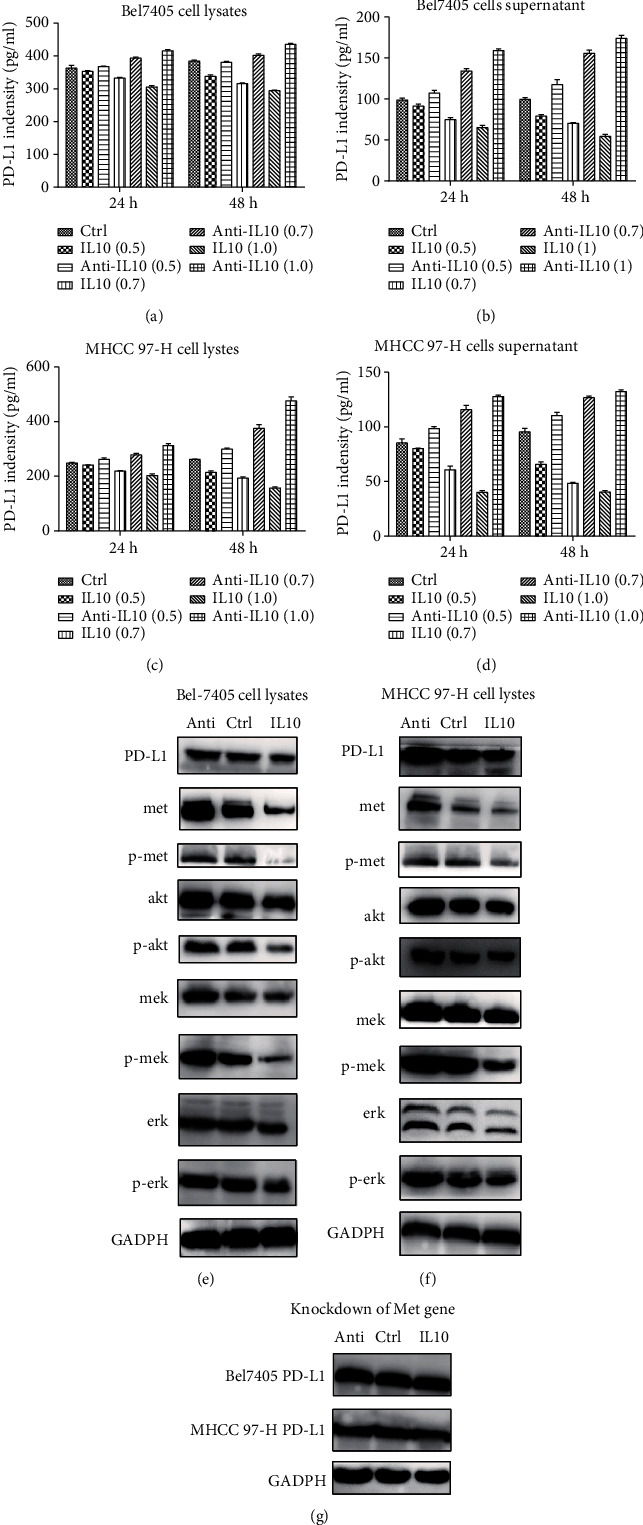
Effects of IL10 or anti-IL10 on PD- L1 expression in LIHC cell lines: (a–d) ELISA experiments for the effects of different concentrations of IL10 or anti-IL10 on the expression of PD-L1 in cell culture supernatant or cell lysates of Bel7405 and MHCC 97-H cell lines; (e, f) Western blots for the effects of IL10 or anti-IL10 on PD-L1, Met signal pathway, and downstream MAPK signaling pathway inBel7405 and MHCC 97-H cell lines. (g) Western blots for the effects of different concentrations of IL10 or anti-IL10 on PD-L1 after the Met gene was knocked down.

Exogenous IL10 reduced the expression level of PD-L1 in LIHC cells. By contrast, addition of anti-IL10 upregulated the expression level of PD-L1 in LIHC cells. PD-L1 expression level is dependent on incubation time and concentration of IL10. A longer incubation time and a higher IL10 concentration produced stronger inhibition effect on PD-L1. Further analysis showed that IL10 downregulated the expression of Met and its downstream signaling targets. Anti-IL10 upregulated Met and its downstream signaling targets (Figures 6(a)–6(f)). Neither IL10 nor anti-IL10 had any effect on the expression level of PD-L1 when the Met gene was silenced (Figure 6(g)).

### 3.5. A Combination of Crizotinib and IL10 Was More Effective Than Crizotinib Alone against LIHC

**Figure 7:**
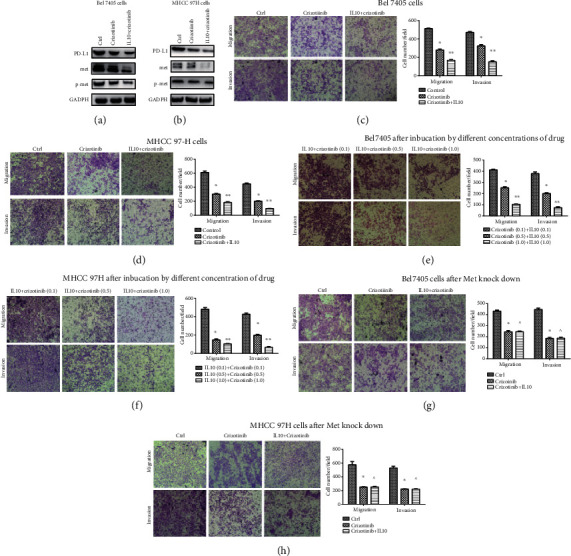
Effects of IL10 and crizotinib on migration and invasion on LIHC cell lines: (a, b) Western blot experiments for the effects of crizotinib and combination of IL10 and crizotinib on expression of PD-L1 and Met signal pathway in two cell lines; (c, d) effects of crizotinib and combination of crizotinib and IL10 on invasion and migration in LIHC cell lines; (e, f) effects of different concentrations of IL10 and crizotinib on invasion and migration in LIHC cell lines (0.1: concentration of IL10 was 0.1 mg/ml and crizotinib was 0.1 *μ*M; 0.5: concentration of IL10 was 0.5 mg/ml, and crizotinib was 0.5 *μ*M; 1: concentration of IL10 was 1 mg/ml, and crizotinib was 1 *μ*M). ^∗^*P* < 0.05 compared with IL10 (0.1)+crizotinib (0.1); ^∗∗^*P* < 0.05 compared with IL10 (0.5)+crizotinib (0.5); (g, h) effects of crizotinib and combination of crizotinib and IL10 on invasion and migration of LIHC cell lines after the Met gene was knocked down in two cell lines. ^∗^*P* < 0.05 compared with the control group. ^∗∗^*P* < 0.05 compared with the crizotinib group. ^*P* ≥ 0.05 compared with the crizotinib group.

**Figure 8:**
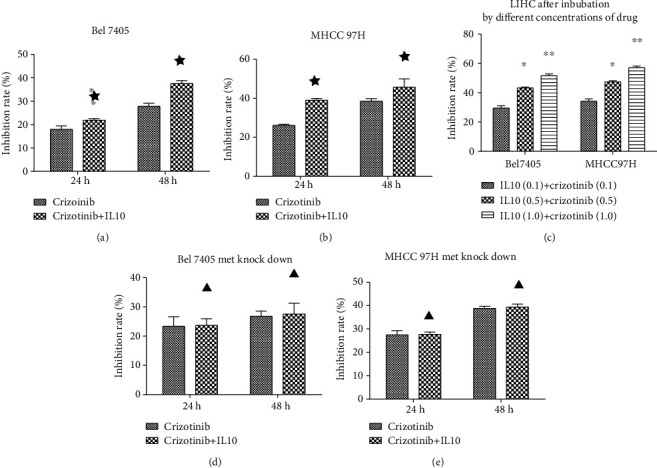
Effects of IL10 and crizotinib on proliferation of LIHC cell lines: (a, b) effect of crizotinib combined with crizotinib and IL10 on proliferation; (c) effects of different concentrations of IL10 and crizotinib on proliferation in LIHC cell lines (0.1: concentration of IL10 was 0.1 mg/ml, and crizotinib was 0.1 *μ*M; 0.5: concentration of IL10 was 0.5 mg/ml, and crizotinib was 0.5 *μ*M; 1: concentration of IL10 was 1 mg/ml, and crizotinib was 1 *μ*M). ^∗^*P* < 0.05 compared with IL10 (0.1)+crizotinib (0.1); ^∗∗^*P* < 0.05 compared with IL10 (0.5)+crizotinib (0.5); (d, e) effect of crizotinib combined with crizotinib and IL10 on proliferation of Bel7405 and MHCC 97-H after the Met gene was knocked down. ★: *P* < 0.05 compared with the crizotinib group; ▲: *P* ≥ 0.05 compared with the crizotinib group.

The inhibitory effect of crizotinib alone on PD-L1 and Met expression in LIHC cells was weaker than that of a combination of crizotinib and IL10 (Figures 7(a) and 7(b)). Similarly, the combination of crizotinib and IL10 was more effective in inhibiting tumor proliferation, invasion, and metastasis compared to crizotinib alone (the concentration of IL10 was 1 mg/ml, and crizotinib was 1 *μ*M) (Figures 7(c) and 7(d); Figures 8(a) and 8(b)). Moreover, a higher drug concentration produced stronger anticarcinoma effect (Figures 7(e) and 7(f); Figure 8(c)). In addition, silencing of the Met gene abolished the effects of the combined treatment indicating that the Met signaling pathway was involved in this process (Figures 7(g) and 7(h); Figures 8(d) and 8(e)).

## 4. Discussion

Bioinformatics is a new field comprising of tools for information storage and analysis. It has accelerated research in life sciences. Here, bioinformatics analysis on TCGA database revealed a correlation between PD-L1 and IL10 in patients with LIHC, which gave us much inspiration for the research.

PD-L1 and IL10 were positively correlated in carcinoma tissues and adjacent tissues. Expression level of PD-L1 and IL10 in adjacent tissues and normal tissues was lower than in carcinoma tissues. These results are consistent with previous reports [36, 37].

IL10 was found to be an independent factor that predicts survival rate of LIHC patients. Notably, low IL10 expression predicted better prognosis. Results showed that low expression levels of PD-L1 and IL10 predicted longer OS and DFS while higher expression levels of PD-L1 and IL10 predicted shorter OS and DFS. These results were consistent with those reported by Chau et al. [38–44].

Some of our data are consistent with TCGA database and have indicated that PD-L1 and IL10 expression levels were positively correlated; furthermore, PD-L1 and CD8 expression levels were also positively correlated. However, some results are different from bioinformatics analysis. Bioinformatics analysis revealed that IL10 did not affect OS and DFS in patients. In our study, the low IL10 expression level group tended to have longer survival periods than those with high IL10 expression levels. This may be caused by the following reasons. First, our results are based on protein expression whereas data of TCGA database relates to gene expression of IL10. Second, the number of samples involved in this study is different from that analyzed in TCGA database. Third, all cases enrolled in this study are Chinese, whereas cases in TCGA database are largely from European and American countries. Ethnic differences may cause the discrepancies observed here.

Currently, drug combinations have become a new trend in cancer treatment. A single treatment and drug lack the therapeutic power to effectively control aggressive tumors. Since the discovery of PD-L1 monoclonal antibody in 2013, its efficacy in cancer treatment has been recognized by both doctors and patients. However, poor clinical response rate has limited its benefits. Therefore, approaches that improve the clinical response rate of PD-L1 should be developed. Many clinical trials and basic studies have demonstrated that the PD-L1 monoclonal antibody combined with chemotherapy, radiotherapy, and other biological treatments provides satisfactory clinical outcomes. We therefore performed this study to explore new methods to enhance the efficacy of the PD-L1 monoclonal antibody.

Previous studies have postulated that IL10 may inhibit the function of immune cells hence inflammation and immune response [29–31, 45]. Lamichhane et al. showed that sustained release of IL10 induced immunosuppression in patients with ovarian carcinoma when PD-L1 was blocked [30]. However, we herein found that IL10 is like a “double-edged sword.” It can activate immune cells under specific microenvironments. In our study, PD-L1 expression was downregulated in LIHC cells treated with exogenous IL10. A combination of crizotinib and IL10 more strongly inhibited PD-L1 expression than crizotinib alone. Further functional experiments revealed that the combination of the two drugs effectively inhibited tumor invasion, proliferation, and metastasis compared to crizotinib alone. Moreover, the effects of the combination were mediated by the met signaling pathway as they were abolished following met gene silencing. This study expands the clinical role of IL10 and provides new ideas for comprehensive treatment of LIHC.

Overexpression of PD-L1 in LIHC cells increased the expression of IL10 and Met. PD-L1 and IL10 were positively correlated in LIHC tissues. Sergio et al. postulated that activation of the HGF/Met signaling pathway can upregulate IL10 expression [46–48]. The HGF/Met signaling pathway plays important roles in human physiological processes. Deregulation of this pathway leads to the occurrence and development of tumors as well as induce drug resistance [49]. We found that PD-L1 also colocalized with Met [50]. Based on our results and those of a previous studies, we postulate that PD-L1, Met, and IL10 form interacting loops, in which Met links PD-L1 and IL10. It has also been postulated that overexpression of PD-L1 activates the Met signaling pathway and upregulates IL10 expression through a positive feedback mechanism. On the other hand, IL10 inhibits the Met signaling pathway and downregulates PD-L1 expression through a negative feedback mechanism.

Nevertheless, our results are limited by several factors. We did not involve animal models to confirm that IL10 enhanced the anticarcinoma effect of PD-L1 inhibitor. The mechanisms of the interaction loops between PD-L1, Met, and IL10 were not explored.

In summary, the relationship between IL10 and PD-L1 in LIHC tissues and cell lines was systematically explored in this study. Our results provide a theoretical basis for the comprehensive targeted treatment of LIHC. Further validations through animal-based studies are needed.

## 5. Conclusions

IL10 downregulated the expression level of PD-L1 and enhanced the efficacy of crizotinib via the Met signaling pathway in the LIHC cells. A combination of IL10 and PD-L1 inhibitor holds great promise as an effective treatment for LIHC.

## Figures and Tables

**Table 1:** Demographic and clinical characteristics death-ligand 1 expression in 100 patients with LIHC.

Characteristic	*n*	PD-L1		*χ* ^2^	*P*
		Low	High		
		25	75		
Sex				0.337	0.562
Male	55	15	40		
Female	45	10	35		
Age (years)				3.922	0.048
<60	32	12	20		
≥60	68	13	55		
Tumor differentiation				12.000	0.001
High	50	20	30		
Moderate/low	50	5	45		
T stage				59.524	<0.001
T1/T2	28	22	6		
T3/4	72	3	69		
N stage				0.000	1.000
N0	52	20	32		
N1	48	5	43		
Tumor location				2.772	0.096
Left half liver	62	12	50		
Right half liver	38	13	25		
Tumor size				0.152	0.696
<5 cm	73	19	54		
≥5 cm	27	6	21		
IL10 status				50.52	<0.001
Low expression	26	20	6		
High expression	74	5	69		
CD8					
Low expression	30	3	27	5.143	0.023
High expression	70	22	48		

*P* < 0.05 was considered the difference that has statistical significance.

**Table 2:** Demographic and clinical characteristics and IL10 in 100 patients with LIHC.

Characteristic	*n*	IL10		*χ* ^2^	*P*
		Low	High		
		33	67		
Sex				1.813	0.178
Male	55	15	40		
Female	45	18	27		
Age (years)				95.528	<0.001
<60	32	8	24		
≥60	68	25	43		
Tumor differentiation				2.216	0.137
High	50	13	37		
Moderate/low	50	20	30		
T stage				42.477	<0.001
T1/T2	28	23	5		
T3/T4	72	10	62		
N stage				100.00	<0.001
N0	52	16	36		
N1	48	17	31		
Tumor location				0.455	0.500
Left half liver	62	22	40		
Right half liver	38	11	27		
Tumor size				100.00	<0.001
<5 cm	73	19	54		
≥5 cm	27	14	13		
PD-L1 status				27.876	<0.001
Low expression	25	19	6		
High expression	75	14	61		
CD8				1.811	0.178
Low expression	30	7	23		
High expression	70	26	44		

*P* < 0.05 was considered the difference that has statistical significance.

**Table 3:** Comparison of PD-L1 expression levels in carcinoma tissues and adjacent tissues.

	*n*	PD-L1 high expression	PD-L1 low expression	*χ* ^2^	*P*
Carcinoma tissue	100	75	25	44.220	<0.001
Adjacent tissue	100	28	72		

*P* < 0.05 was considered the difference that has statistical significance.

**Table 4:** Comparison of IL10 expression levels in carcinoma tissues and adjacent tissues.

	*n*	IL high expression	IL low expression	*χ* ^2^	*P*
Carcinoma tissue	100	67	33	20.488	<0.001
Adjacent tissue	100	35	65		

*P* < 0.05 was considered the difference that has statistical significance.

## Data Availability

All the data were available upon appropriate request.

## Supplementary Materials

Supplementary Materials The supplementary materials mainly describe the experimental procedures in detail, such as immunochemistry, RT-PCR, and Western blotting. The supplementary materials mainly include three tables. Table S1: the scoring criteria for immunohistochemistry. Table S2: primers for RT-PCR. Table S3: siRNA sequence of Met and PD-L1. Furthermore, specific process for ELISA, cell culture, transwell, and CCK8 is also described in supplementary materials.
