# Supplementary material for: Relationship between IL10 and PD-L1 in Liver Hepatocellular Carcinoma Tissue and Cell Lines
Source: Biomed Res Int. 2020 Jul 16;2020:8910183. doi: 10.1155/2020/8910183 (PMC7381951; doi:10.1155/2020/8910183)
Supplement: Supplementary Materials — The supplementary materials mainly describe the experimental procedures in detail, such as immunochemistry, RT-PCR, and Western blotting. The supplementary materials mainly include three tables. Table S1: the scoring criteria for immunohistochemistry. Table S2: primers for RT-PCR. Table S3: siRNA sequence of Met and PD-L1. Furthermore, specific process for ELISA, cell culture, transwell, and CCK8 is also described in supplementary materials. [file 8910183.f1.doc]

**Supplementary material**

**Immunochemistry**

Tissue sections were dewaxed with xylene and rehydrated with ethanol. Boiled EDTA was used for antigen repair. 3% bovine serum albumin (BSA) was used for blocking after which the sections were incubated with the corresponding primary antibody overnight. Both PD-L1 and IL10 antibodies were purchased from American Novus Company(Littleton, Colorado, USA) (dilution ratio was 1: 200). CD8 antibody was purchased from Dako Company(Copenhagen,Denmark) (dilution ratio was 1:50). Tissue sections were then washed with PBS and then incubated with a secondary antibody(Maixin, Fuzhou, Fujian, China) for 30 minutes. DAB solution was then used to stain the sections after washing. Hematoxylin was used for counterstaining. The tissues was finally dehydrated with Ethanol and xylene and examined under a microscope.

The staining intensity was evaluated according to the scoring criteria presented in TableS1. It was then multiplied with the rate of positive cells to obtain the final semi-quantitative score . All IHC images were evaluated by two experienced pathologists. Scores less than 8 were defined as low expression while those above 8 were defined as high expression.

**Table S1. Score for cell intensity or the rate of positive cells**

| Cell intensity | |  | Positive cells | |
| --- | --- | --- | --- | --- |
| Score | Degree of staining |  | Score | The rate of positive cells |
| 1 | Negative staining |  | 1 | 0-25% |
| 2 | Moderate staining |  | 2 | 26-50% |
| 3 | Strong staining |  | 3 | 51-75% |
|  |  |  | 4 | >75% |

**Quantitative real time PCR**

Liver carcinoma tissues and adjacent tissues were first homogenized and the Trizol reagent used to extract total RNA from the tissues. RNA quality and quantity were the determined using NanoDrop TM3300 Fluorospectrometer(Thermo Scientific,Waltham，American). An RT-PCR kit purchased from Takara Biotechnology(Tokyo,Japan)was used to detect the expression levels of PD-L1, IL10 and Met mRNA in carcinoma tissues and adjacent tissues. Primers were designed by Shanghai Sangon Company(Shanghai,China) (Table S2). The reverse transcription and PCR reactions were carried out on the Biosystems 7500 Fast Real-Time PCR system(Thermo Scientific,Waltham，American).

**Table S2. Primers for RT-PCR**

| Gene | Sequence |
| --- | --- |
| IL-10 | Forward 5’-CTT CGA GAT CTC CGA GAT GCC TTC-3’ |
|  | Reverse 5’-ATT CTT CAC CTG CTC CAC GGC CTT-3’ |
| Met | forward 5’-GGT TCA CTG CAT ATT CTC CCC-3’ |
|  | Reverse 5’-ACC ATC TTT CGT TTC CTT TAG CC-3’ |
| PD-L1 | Forward 5’-ACT GGC ATT TGC TGA ACG-3’ |
|  | Reverse 5’-TCC TCC ATT TCC CAA TAG AC-3’ |
| GADPH | Forward 5’-TGA CTT CAA CAG CGA CAC CCA-3’ |
|  | Reverse 5’-CAC CCT GTT GCT GTA GCC AAA-3’ |

**Cell culture**

LIHC cell lines Bel7405 and MHCC 97H were sourced from the Cell Research Center, Third Affiliated Hospital of Soochow University.They were placed in a six-well plate (3×10^4^-10^5 /^well) containing DMEM(Hyclone, Logan City, Utah, USA) comprised of 10% fetal bovine serum(FCS; Gibco, Thermo-Fisher Scientific, Grand Island, NY, USA). They were then incubated in a cell incubator at 37°C with 5% carbon dioxide concentration. Different concentrations of exogenous IL10 and anti-IL10 were added to the cells when the cell density reached 75% (0.5u/well to 1u/well). Both IL10 and anti-IL10 were purchased from the American Creative Biolabs(New York,USA).

**Construction siRNA**

The SiRNA sequence was designed by Shanghai Gene Pharma biological company(TableS3). We used customized siRNA (Gene Pharma Company, Shanghai, China) and configured an X-treme GENE siRNA Transfection Reagent - siRNA plasmid mixture, which was then added to the cells to be transfected. RT-PCR was used to detect efficiency 24 hours after transfection. Transfection efficiency was confirmed to be above 70% before proceeding with subsequent experiments.

**Table S3. siRNA of Met and PD-L1**

**ELISA**

| siRNA | Sequence |
| --- | --- |
| siMet | Sense:5’-CTC ATT TGG ATA GGC TTG TAA-3’ |
|  | Anti-sense:5’-TTA CAA GCC TAT CCA AAT GAG-3’ |
| siPD-L1 | Sense :5’- CAG AAA GAU GAG GAU AUUU-3’ |
|  | Anti-sense: 5’-AAA UAU CCU CAU CUU UCUG -3’ |

IL10 expression level in cell cultures supernatants of siPD-L1 and LV-PD-L1 was determined using the IL10 hypersensitive ELISA kit from Multi Sciences company(Hangzhou,China). The sample was diluted and then used to test for immune response following the manufacturer’s instructions. The Thermo Scientific full-wavelength scanning reader was used to measure the OD value of the samples at 560 nm.

LIHC cell lines were cultured with varying concentrations of exogenous IL10 and anti-IL10 for 24 to 48 hours. Cell culture supernatant and cell lysates was then taken for ELISA tests. The PD-L1 ELISA kit sourced from the American R&D biological company (Minneapolis,MN,USA)was used for this experiment. The sample was diluted and then used to test for immune response following the manufacturer’s instructions. The Thermo Scientific full-wavelength scanning reader was used to measure the OD value of the samples at 560 nm.

**Transwell and CCK8**

The transwell chamber of the American Coring Company(New York, USA) was used to detect metastasis and invasion of cells. The upper chamber without glue at the bottom was used to detect the metastasis of cells while the upper chamber with glue at the bottom was used to detect the invasion of cells. About 2000 to 10000 cells were seeded on each well. Serum-free medium was placed in the lower chamber after which Crizotinib or Crizotinib combined with IL10 were added. Crizotinib was purchased from American CST Biotechnology(Danvers, MA, USA). After 24 or 48 hours, the culture medium was discarded and cells fixed with formaldehyde. The cells were then stained with crystal violet and counted under the microscope.

In CCK8 experiment, 96-well plates were used. 2000 LIHC cells seeded in every well. Crizotinib or Crizotinib combined with IL10 was then added to the cell medium. After 24 to 48 hours, 100ul CCK8 solution (Dojindo Company,Tokyo，Japan) was added to each well and incubated for 4 hours at 37℃. The Thermo Scientific full-wavelength reader was then used to measure the OD values of the samples. The inhibition rate was calculated using the following formula: Inhibition rate = [(Control well-experimental well)/(Control well-blank well)]×100%.

**Western blotting**

LIHC cells were cultured in exogenous IL10 or anti-IL10. Cellular proteins were then extracted using a protein extraction agent(KeyGen Biotech, Nanjing, Jiangsu, China). Protein concentration was measured using bicinchoninic acid assay (BCA，Pierce, Thermo-Fisher Scientific, Waltham, MA, USA)). SDS-PAGE electrophoresis was performed using a 10% precast gel sourced from Beyotime Biotechnology（Shanghai,China）. The protein bands were then transferred onto a PVDF membrane after methanol activation. The PVDF membrane was first blocked with 3% BSA at room temperature for 24 hours. .It was then incubated with the corresponding primary antibody and secondary antibody. PD-L1 expression levels,Met and it its downstream signaling targets(Met,phospho-Met,akt, phospho-akt, Mek, phospho-Mek, Erk, phospho-Erk) were analyzed. In addition, we performed Western blotting on LIHC cells lines that over-expressed or knocked down in the expression of the PD-L1 gene to detect differences in IL-10 and Met expression levels. Subsequently, Crizotinib or Crizotinib combined with IL-10 were added to the cells. PD-L1 and Met were analyzed.

For tissue experiments, liver specimens were minced and homogenized. Proteins were extracted from tissue using protein extraction agent. The concentration of proteins was detected using BCA method. The expression levels of PD-L1, IL10 and Met in carcinoma tissues, adjacent tissues and normal tissues were detected using western blotting.

PD-L1 and IL10 primary antibodies were purchased from American Novus Biotechnology(Littleton, Colorado, USA). The remaining primary antibodies for western blotting were purchased from American CST Biotechnology (Dilution ratio was 1:1000). Secondary antibodies were purchased from American CST Biotechnology (Dilution ratio was 1：10000).
